# Supplementary material for: CD44s Induces miR-629-3p Expression in Association with Cisplatin Resistance in Head and Neck Cancer Cells
Source: Cancers (Basel). 2020 Apr 1;12(4):856. doi: 10.3390/cancers12040856 (PMC7226407; doi:10.3390/cancers12040856)
Supplement: Supplementary file 1 [file cancers-12-00856-s001.pdf]

Supplementary Materials

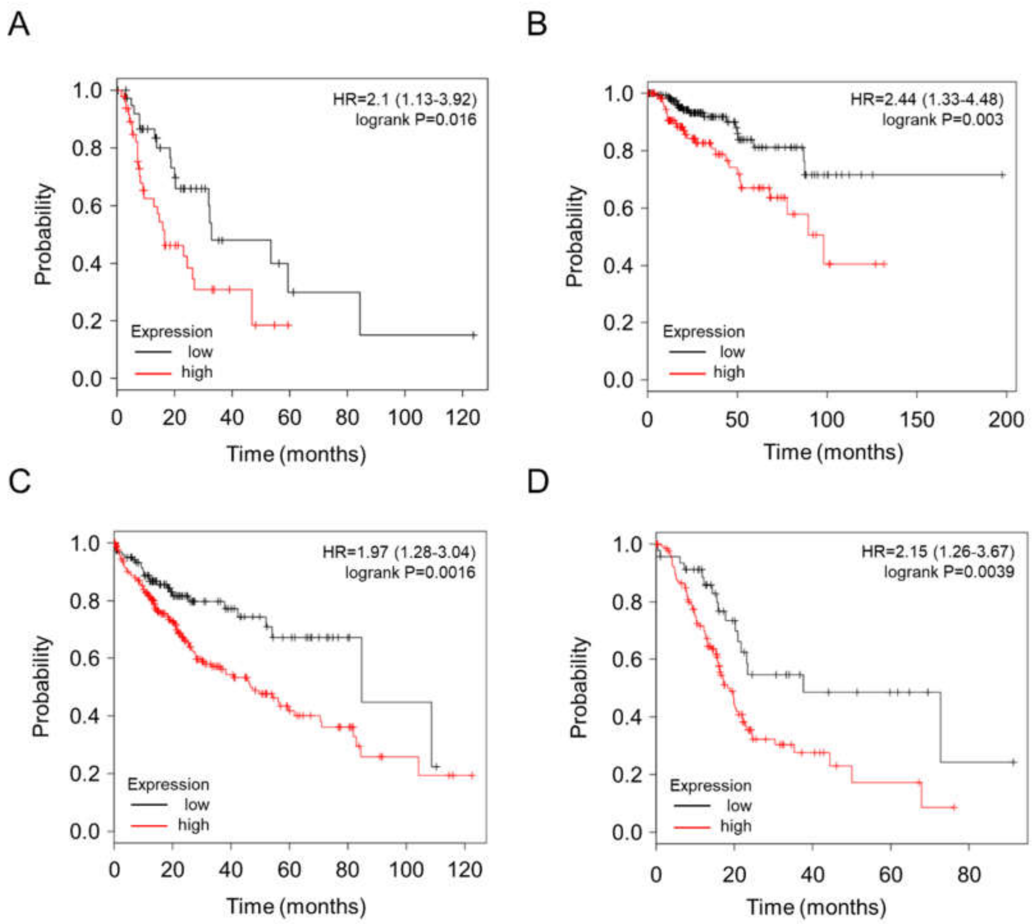

Figure S1: Kaplan-Meier plots of the probabilities of survival in cancer types other than head and neck cancer. (A) Esophageal adenocarcinoma (B) kidney renal papillary cell carcinoma (C) liver hepatocellular carcinoma (D) pancreatic ductal adenocarcinoma.

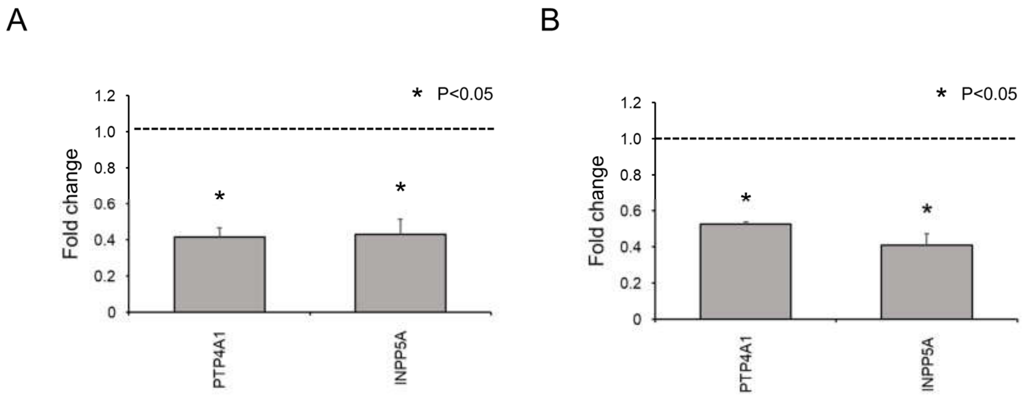

Figure S2: Quantitative RT-PCR analysis of HSC-3 and HSC-4 cells. (A, B) Quantitative RT-PCR analysis of 2 genes in HCS-3 and HCS-4 cells transfected with miR-629-3p. Expression levels were normalized to β-actin, and relative expression was calculated using the comparative CT method.

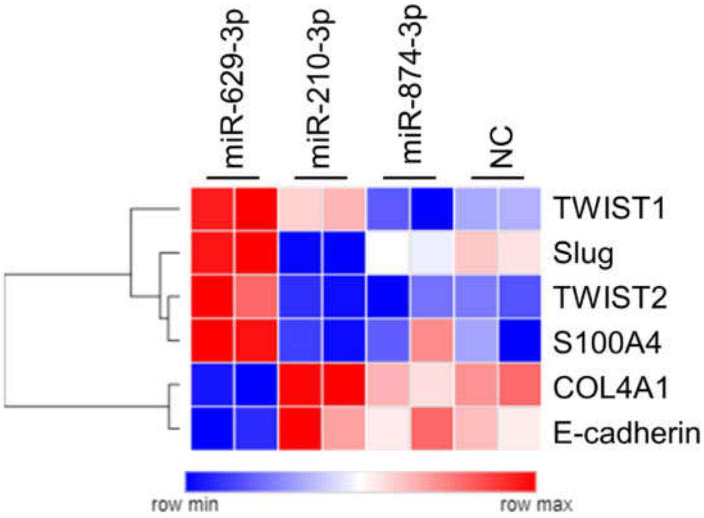

Figure S3: Figure R2 EMT marker expression changes in miR-629-3p transfected SAS cells. EMT regulators, TWIST1, Slug and TWIST2 were upregulated in miR-629-3p transfected SAS cells. Epithelial markers, COL4A1 and E-cadherin were downregulated, and a mesenchymal marker, S100A4 was upregulated in miR-629-3p transfected SAS cells.

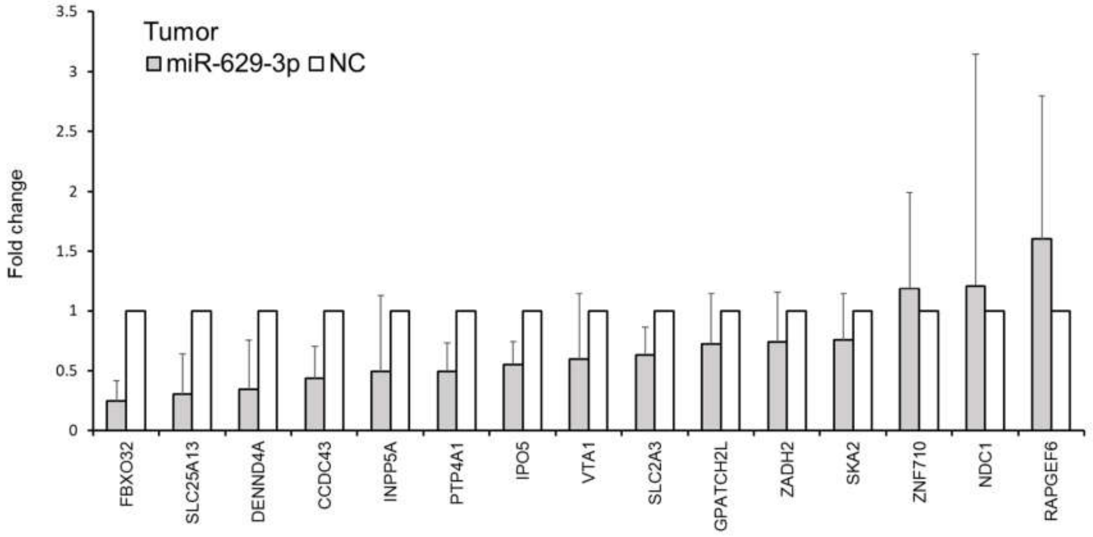

Figure S4: Downregulation of the putative miR-629-3p targeted genes in xenograft tumors of stable miR-629-3p expressing SAS cells. The expression levels of genes were normalized with β-actin, and the expression levels of negative control tumors set to 1.0. Undetectable genes in tumor samples were excluded from 19 genes. Each n = 6.

Table S1. Primer sequences for quantitive RT-PCR.

Primer name	Nucleotide sequence (5' - 3') / TaqMan Assay ID
SLC2A3 Fw	accggcttcctcattacctt
SLC2A3 Rv	aggctcgatgctgttcatct
LBH Fw	atctgagatcggccaagatg
LBH Rv	cttctgtgggttccaccact
PTP4A1 Fw	ccagctcctgtggaagtcac
PTP4A1 Rv	aaggccaatcaagaacatgg
APOL4 Fw	actagcgatgaagcctggaa
APOL4 Rv	agcctctgtggaccttttca
GPATCH2L Fw	ggaagcgtcgttctgacttc
GPATCH2L Rv	ggtgtcgtttggctaccatt
ZNF710 Fw	tacaacctggtgacgcacat
ZNF710 Rv	cagctgtagggcttgacctc

IPO5 Fw<br>IPO5 Rv	tgcctcttctcctggagtgt<br>tgcactttgcaaaagaatgc
MYOCD Fw<br>MYOCD Rv	ctcggcttcctttgaacaag<br>cttcccagagaatccatcca
RAP2A Fw<br>RAP2A Rv	gagcttccaggacatcaagc<br>ctgcctcacaatttctgcaa
NDC1 Fw<br>NDC1 Rv	tgtttcctgttcaaccacca<br>ttggctgaggctgaaaactt
STMN1 Fw<br>STMN1 Rv	aaggatctttccctggagga<br>tgtgcctctcggttctcttt
LRRC58 Fw<br>LRRC58 Rv	gctgcacggaccattaagat<br>ggaagtggagctgtgagagg
INPP5A Fw<br>INPP5A Rv	ttgcagactgtgcctttgac<br>aaaccttctcgaatcgctga
FBXO32 Fw<br>FBXO32 Rv	tcacagctcacatccctgag<br>agacttgccgactctttgga
RAPGEF6 Fw<br>RAPGEF6 Rv	ccatcaggaagaagggacaa<br>tgaggagatgcaggaggact
ERBB4 Fw<br>ERBB4 Rv	tttcgggagtttgagaatgg<br>gaaactgtttgccccctgta
C15orf48 Fw<br>C15orf48 Rv	agcctcatctttcgctgtgt<br>tggtcacccttttggacattt
DENND4A Fw<br>DENND4A Rv	gtcagggctctgaaaacagc<br>tgcatttcaaaaagcactcg
RAB12 Fw<br>RAB12 Rv	atttgccgaaatggatgaag<br>tggcacttgcttcacagaac
TMEM212 Fw<br>TMEM212 Rv	aagccttggttcacaggatg<br>ccaggagagcagattccaag
AFF1 Fw<br>AFF1 Rv	tagtgtccaccaccagtcca<br>gtggtctccgtcagctcttc
CCDC43 Fw<br>CCDC43 Rv	aagagaagctggacgctctg<br>ggcaatggcctgtacttcat
FAM76A Fw<br>FAM76A Rv	cgcccaaaccttgtcagtat<br>tcttctgaaggacccgtttg
ACSM2A Fw<br>ACSM2A Rv	cggggaatcaaagatgaaga<br>acaaatgccttcaccacctc

SOX11 Fw<br>SOX11 Rv	tgatgttcgacctgagcttg<br>tagtcggggaactcgaagtg
FRAT2 Fw<br>FRAT2 Rv	gctctcgggaaacctcatc<br>tcagagcaaggagcctgag
ZBTB21 Fw<br>ZBTB21 Rv	gctggctttgaagagaccac<br>tggctatccatcgacaatga
VTA1 Fw<br>VTA1 Rv	gcaatgcagactggaatgaa<br>ttttgtgaaatcgtccagca
ZADH2 Fw<br>ZADH2 Rv	agggcttcttcctgaaccat<br>ttgacagcacggaatatgga
PPP1R3B Fw<br>PPP1R3B Rv	ggttcagacagggacacgtt<br>cttccgaactggtcaaagga
GAS2 Fw<br>GAS2 Rv	aagatcccatgcaaaaccag<br>ggccaagctctagcagacag
SLC25A13 Fw<br>SLC25A13 Rv	tgcaagcattgagaaaaacg<br>ttctcctttgccagctttgt
SKA2 Fw<br>SKA2 Rv	ccgctttaaaccagttgctg<br>ctctgccgcagttttctctt
TPM3 Fw<br>TPM3 Rv	cctgcaaaagctggaagaag<br>tctgcctcttctgcaatgtg
hsa-miR-629	Assay ID : 001562
RNU6B	Assay ID : 001093
